# The Effect of Emotional Neglect on Cyberbullying among Rural Chinese Left-behind Adolescents—Mediating Role of Social Anxiety

**DOI:** 10.3390/children10061055

**Published:** 2023-06-13

**Authors:** Xinran Hu, Bin Xiao

**Affiliations:** 1 School of Agricultural Economics and Rural Development, Renmin University of China, Beijing 100872, China; huxr1199@ruc.edu.cn; 2 School of Humanities and Law, Northeastern University, Shenyang 110819, China

**Keywords:** emotional neglect, cyberbullying, social anxiety, rural left-behind adolescents

## Abstract

Background: Cyberbullying is a globally shared youth problem—a problem of the interpersonal conflicts and contradictions that emerge during the socialization of adolescents. In particular, the issue of cyberbullying among rural left-behind adolescents needs to be given high priority. However, previous studies have paid little attention to how emotional neglect and social anxiety affect the cyberbullying behavior of rural left-behind adolescents. Therefore, this study was based on cognitive-behavioral theory to investigate the relationship between emotional neglect, cyberbullying, and social anxiety. Methods: This study used the Emotional Neglect Scale, the Cyberbullying Scale, and the Social Anxiety Scale to conduct an anonymous online survey of 1429 rural left-behind adolescents in China. Results: (1) Emotional neglect, social anxiety, and cyberbullying showed a two-way positive correlation. (2) The direct effect of emotional neglect on rural left-behind youth cyberbullying was significant (β = 0.14, *p* < 0.00). (3) Social anxiety showed a partial mediating effect in the process of emotional neglect affecting rural left-behind youth cyberbullying, with a mediating effect of 26.32%. Conclusions: The results have positive implications for improving relevant policies and constructing mechanisms for protecting the rights and interests of rural left-behind adolescents.

## 1. Introduction

Along with rapid industrialization and urbanization in China, many surplus laborers have moved from rural to urban areas, forming a massive group of “migrant workers”. The China Bureau of Statistics reported in April 2023 that the total number of migrant workers in China reached 295.6 million in 2022, including 172 million migrant workers outside of rural areas, accounting for 34.6% of China’s total rural household population [1]. Although rural areas contribute to increasing family income and improving living conditions, a large number of parents are separated from their children, resulting in the emergence of a particular group—left-behind youth [2]. The definition of left-behind adolescents differs slightly from study to study but includes aspects such as residence status, age, and time of parental migration. It has been generally agreed that those secondary school students aged 13–18 who live and study in rural areas, with one or both parents who have migrated to other regions for work for more than six months, are defined as left-behind adolescents [3,4].

During the growth of left-behind youth, the absence of parental companionship and education has, to a certain extent, brought adverse physical and psychological effects, causing social problems. Internet technology is an important attraction for rural left-behind youth, and when left-behind youth are addicted to the Internet, they tend to develop recognition of the virtual world and lack of control over their own behavior, which, in turn, can contribute to phenomena such as cyber-attacks and cyber-addiction [5,6]. Cyberbullying is a new type of abusive tactic created in the Internet era, which refers to the repeated dissemination of false, embarrassing, or hostile information about another person over some time via the Internet or mobile technology, causing negative emotions such as uneasiness or embarrassment in the other person [7,8]. Specific types of cyberbullying include deception, humiliation, and unwanted contact [9]. Adolescents are at a time when they are shaping their emotional value system and are susceptible to the social environment and emotional processing. During the critical period of adolescence and growth and development, left-behind teenagers often show rebellion, stubbornness, and capriciousness in their psychological changes [10]. The anonymity and virtual nature of the Internet provide a venue for venting negative emotions [11]. In addition, cyberbullying becomes, to some extent, a way for victims of school violence to retaliate for the traditional bullying they have experienced offline [12]. As a result, cyberbullying has become a growing problem among adolescents. For rural adolescents left behind, the lack of effective discipline and proper guidance of values may exacerbate the occurrence of cyberbullying. On the one hand, the lack of effective discipline can reduce the social normative level for adolescents to discipline their own online behavior. On the other hand, the lack of positive guidance can also result in the inability of adolescents to establish correct perceptions in terms of Internet use.

Under cognitive-behavioral theory, with the help of the stimulus-stress analysis framework, it has been pointed out that dependence on Internet use occurs when individuals face significant stressors and specific events. When individuals have adverse emotions and are influenced by stressors, they are prone to Internet addiction and unintended consequences such as cyberbullying. However, few studies on cyberbullying research have included the particular group of Chinese rural left-behind adolescents as an object of study, and few scholars have included emotional neglect. This environmental factor predisposes individuals to significant stressors from a research perspective. Therefore, this paper provides insight into the mechanisms that influence these two factors on the cyberbullying behavior of rural left-behind adolescents.

Emotional neglect often causes individuals to develop emotional- and cognitive-level biases, meaning that emotional neglect often leads to more negative emotional experiences [13]. Some studies have pointed out that emotional neglect behaviors tend to occur more commonly in rural groups of left-behind adolescents [14,15]. Due to China’s urban-rural dichotomy, most migrant workers are unable to bring their children with them, and most school-age youth are left behind in rural areas, which leads to a chronic lack of companionship and emotional absence, essentially increasing the probability of emotional neglect. Emotional neglect has been defined in the literature as the persistent indifference of parents to the psychological and emotional needs of their children [15] and the inability to give them the love they deserve [16]. It has been generally accepted that emotional neglect can have a range of adverse consequences on adolescents’ emotions and behaviors [13,17,18], including adolescent depression [19], internalizing traits such as anxiety [20], posttraumatic stress disorder [21], and juvenile delinquency [22], aggression [23], and other externalizing characteristics. However, few studies have addressed the impact of emotional neglect on cyberbullying, especially in studies of rural left-behind youth. In addition, to better explain why this paper includes two variables—emotional neglect and social anxiety—in the same framework of cyberbullying research, this paper introduces Davis’ cognitive-behavioral theory, which divides the triggers that produce cyberbullying behavior into two categories: distal and proximal inducements. This theory reflects social anxiety as a proximal inducer, while emotional neglect is a distal inducer. Applying this theory provides the theoretical basis for the research framework of this paper [24].

Social anxiety refers to an individual’s excessive fear of being negatively evaluated by others during social interactions [25]. It is concentrated in the individual’s emotions and behaviors, such as nervousness, fear, and avoidance of interactions with others and social situations [26]. It has been noted that social anxiety is one of the most prominent problems manifested by the group of adolescents left behind in China [14], which may lead to problems such as depression, substance use disorders, and poor career development in adulthood [27]. According to the social compensation hypothesis, social anxiety causes adolescents to increase their dependence on the online world to replace their social fear of the natural world [28], leading to their addiction to the Internet. Further, it has been suggested that social anxiety contributes to the development of cyber-aggressive behavior in adolescents [29]. It has been clearly stated in the literature that traumatic experiences such as emotional neglect can lead to social anxiety in adolescents [30]. It has also been shown that socially anxious people develop negative social cognitive biases, which can lead to negative behaviors such as aggression, irritability and bullying [31]. The association between emotional neglect, social anxiety, and cyberbullying is rarely mentioned in related studies, so studying the relationship between these three factors is relevant. At the same time, this paper also broadens the research model on the causal factors of cyberbullying behavior in specific groups. It has practical implications for studying a particular group of rural left-behind adolescents.

### 1.1. The Relationship between Emotional Neglect and Cyberbullying

As a model of emotional abuse, emotional neglect is more common among adolescents [32]. The lack of care and high vulnerability to emotional neglect among rural left-behind adolescents dramatically increases the probability of their indulgence in cyberbullying. The characteristics of cyberbullying, such as ambiguous management, the severe difficulty of discipline, and not easily being detected, invariably provide potential conditions for adolescents to commit cyberviolence. According to Maslow’s Hierarchy of Needs theory, after physiological and safety needs are satisfied, individuals will pursue emotional needs, and if emotional needs are not satisfied, negative emotions such as low self-esteem and depression will occur. Some scholars have pointed out that individuals who suffer from severe emotional neglect are significantly more likely to engage in antisocial behaviors [33], such as violent tendencies, dating crimes, etc. Cyberbullying is a new form of violence, and it is conceivable that emotional neglect may lead to cyberbullying to a certain extent.

Studies on the phenomenon of cyberbullying have pointed out that a lack of self-control [34], antisocial personality [35], and moral disengagement [36,37] are important influences leading to the phenomenon of adolescent cyberbullying factors [11], and emotional neglect often brings these negative factors to adolescents. Some literature has examined the relationship between childhood emotional trauma in adults, including emotional decay and dissociative trauma, as well as cyberbullying, and have concluded that there is a direct or indirect relationship [38,39]. Conversely, the warmth given by parents [40] and family support for adolescents can all reduce the probability of and protect adolescents from cyberbullying.

Based on existing research on the damaging psychological trauma of emotional neglect, we proposed that:

**Hypothesis** **1.** *Emotional neglect in rural left-behind adolescents may be positively associated with cyberbullying behavior.*

### 1.2. The Mediating Role of Social Anxiety

Regarding the causes of social anxiety, it can be divided into three categories: family factors [27], individual factors [41], and genetic factors [42], where family factors mainly refer to the relationship between members, communication status, and the degree of family warmth. Emotional neglect, to some extent, maps family factors and increases the probability of social anxiety in individuals. Some studies have confirmed that childhood emotional neglect positively predicts social anxiety in adolescence and adulthood [43]. It can be inferred that rural left-behind adolescents will have a higher probability of social pressure than non-left-behind adolescents.

Some studies have indicated that social anxiety is an identified risk factor for adolescent cyberbullying [29]. Cognitive-behavioral theory suggests that socially anxious individuals find it difficult to establish good interpersonal relationships in real life. Thus, they prefer online social interactions [44,45] and view the Internet as an unsupervised virtual world for venting bad feelings, which, to some extent, increases the probability of cyberbullying. Some studies have shown that social anxiety is a predictor of the development of cell phone addiction in adolescents and adults [46], suggesting that social anxiety is positively associated with Internet addiction and cyberbullying. In addition, by exploring the relationship between social anxiety and cyberbullying among college students, some scholars have concluded that social anxiety and cyberbullying are positively related to victimization and crime [31].

Based on the existing literature, the second research hypothesis of this paper was proposed:

**Hypothesis** **2.** *Social anxiety may mediate the mechanism of emotional neglect on cyberbullying among rural left-behind adolescents.*

**Figure 1:**
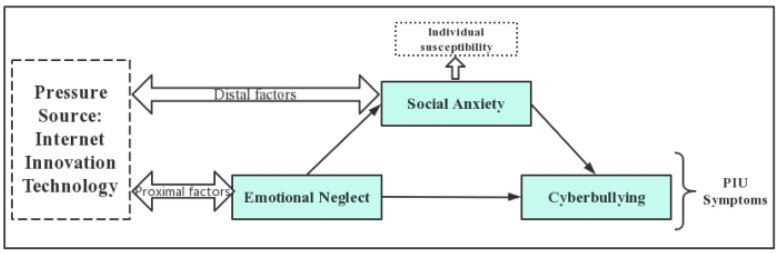
Theoretical framework diagram.

This study investigated (1) whether emotional neglect affects rural left-behind adolescents’ cyberbullying behaviors, and (2) the mediating mechanism of social anxiety in the influence of emotional neglect on rural left-behind youth cyberbullying behavior. Based on cognitive-behavioral theory, this paper incorporated the distal causative factor of emotional neglect and the proximal causative factor of social anxiety into the analysis of cyberbullying in the PIU cognitive-behavioral model. It integrated a mediating model to examine the effects of emotional neglect and social anxiety on cyberbullying among left-behind youth in rural China. Specifically, this paper took the behavior of cyberbullying among rural left-behind adolescents as the main content of research. At the same time, emotional neglect was taken as the emotional variable, and social anxiety was taken as the cognitive variable of this model. The characteristics and variations of cyberbullying behavior were reflected through the dual influence of cognitive variables and emotional variables. The following is a diagram of the theoretical framework of this study (Figure 1).

## 2. Research Methodology

### 2.1. Research Design

The empirical data were collected through the online survey platform “Questionnaire Star” and “School Data Platform Collection Network”. An online questionnaire link was developed and shared with rural schools in eastern, northern, western, southern, and central China. This research was a cross-sectional study. The questionnaire survey covered a wide range of rural areas, reflecting the population’s breadth and the survey’s regional value. During the research process, the respondents were defined as rural left-behind adolescents attending the junior to senior high-school level [3,47].

The questionnaire section of this paper first explains the purpose, the meaning, and the importance. Next, it provides the questionnaire content collection related to participant information. It emphasizes that all the collected questionnaire information reflects the principles of confidentiality, ethics, and science. The second part includes the contents of the basic personal profile and family profile questionnaire, as well as the contents of the Cyberbullying Scale, the Emotional Neglect Scale, and the Social Anxiety Scale. To improve the quality and validity of the questionnaire responses, the participants were set to take more than 10 min to complete the questionnaire. A total of 1830 respondents were recruited, and the survey generated 1429 complete and valid data, with a reasonable proportion of 78.09%. Among them, 693 were male (48.50% of the total), 736 were female (51.50% of the total), 668 were junior high-school students (46.75% of the total), 761 were high-school students (53.25% of the total); 563 (39.4%) were teenagers whose parents both worked outside the home, 586 (41.01%) were teenagers whose fathers worked outside the house, and 280 (19.59%) were teenagers whose mothers worked outside the home. The average age of the subjects was 14.16 ± 1.35 years. The questionnaire process ensured that the teachers and the students had the right to information and consent and that the results were kept confidential. The Academic Ethics Committee of the Renmin University of China and Northeastern University approved the study.

### 2.2. Research Tools

#### 2.2.1. Cyberbullying Scale

The cyberbullying subscale developed by Erdur and Kavsut [48] was used, which consisted of 18 questions. The scale was used to represent the number of times cyberbullying was committed and the motivation for doing so. The scale was scored on a 4-point Likert scale from “1–4”, where “1” means no cyberbullying ever and “4” means cyberbullying more than five times. The higher the score, the higher the level of cyberbullying the individual committed. The scale has been shown to have good reliability and validity in the Chinese subject population [49]. In this study, Cronbach’s alpha coefficient for this subscale was 0.886, and the KMO for the cyberbullying scale was 0.897. The model fit indicators also yielded CFI = 1.00, GFI = 0.96, RMSEA = 0.03, and RMR = 0.06, all of which met the criterion of a good fit [50].

#### 2.2.2. Emotional Neglect Scale

The Emotional Neglect Scale of the Childhood Trauma Questionnaire (CTQ-SF) developed by Bernstein et al. [51] and Zhang [52] was used to form the Adolescent Emotional Neglect Scale, which consisted of 8 questions. The scale primarily represents the extent to which the families emotionally neglected adolescents. For example, “I felt loved by my family at the time” and “My family cared for each other at the time”. A 5-point Likert scale (from “1 = never” to “5 = always”) was used, and the scores were reversed. The higher the score on the questionnaire, the greater the experience of emotional neglect during adolescence. The Cronbach’s alpha coefficient for this scale in this study was 0.879, and the KMO value for the emotional neglect scale was 0.883. The model appropriate indicators also yielded CFI = 0.994, GFI = 0.931, RMSEA = 0.02, and RMR = 0.034, all of which met the criterion of a good fit [50].

#### 2.2.3. Social Anxiety Scale

The Social Anxiety Scale (SAS-SMU) developed by Alkis et al. [53] and Li et al. [54] was used, which consisted of 15 items. The scale was mainly used to represent adolescents’ social anxiety levels. The scale uses a 5-point Likert scale (from “1 = not at all” to “5 = fully”), and the higher the score, the higher the level of social anxiety. In this study, Cronbach’s alpha coefficient of the scale was 0.913, and the KMO of the social anxiety scale was 0.907. The model fit indicators also yielded CFI = 0.991, GFI = 0.899, RMSEA = 0.03, and RMR = 0.029, all of which met the criterion of a good fit [50].

### 2.3. Data Analysis

First, the standard method bias test was performed in this study. Second, 95% confidence intervals (CIs) and the odds ratios (ORs) for each regression model are presented. The tests were two-sided, and statistical significance was determined by *p* < 0.05. All analyses were performed using SPSS 24.0. Third, the structural validity of the scales was validated, and models were constructed using Mplus 7.4 to test for common methodological biases. Mediating effects were tested using the PROCESS Macro plug-in. Preacher and Hayes suggested that bias-corrected 95% confidence intervals based on 5000 samples assess all indirect effects in the model [55]. The significance of the mediating variable was reflected by not including zero in the confidence interval.

## 3. Results

### 3.1. Common Method Bias Test

The research method used in this study included self-statement scales, which may be subject to common method bias. To control for common methodological biases, the author administered the questionnaire so that subjects were informed of the anonymity and confidentiality of their answers, and the questionnaire included reverse and polygraph questions. In addition, Harman’s one-way method was used for testing. The questions were subjected to exploratory factor analysis. It was found that the KMO value was 0.913, the *p*-value of Bartlett’s spherical test was less than 0.001, and the variance explained by the first factor was 29.56%, which was lower than the critical criterion of 40%, so there was no common severe method bias in this study.

### 3.2. Variable Setting

This section sets and describes the variables used in the regression analysis, and the primary setting criteria are shown in Table 1. On the one hand, the cyberbullying, social anxiety, and emotional neglect variables were included in the regression model. The values taken were the mean scores of the scale questions, which represent the average of the scores of these scales. On the other hand, the importance of the remaining variables, such as gender, only child or not, and study stage, were dummy variables expressed as continuous scores. In addition, the table reflects the mean and standard deviation of all variables.

### 3.3. Regression Analysis of Relevant Variables

Based on the classification results, the effects of demographic variables on emotional neglect, cyberbullying, and social anxiety were examined. Multinomial logistic regressions were conducted with emotional neglect, cyberbullying, and social anxiety potential as dependent variables, and gender, whether they were only child, and learning stage as independent variables, respectively, to obtain OddsRatio coefficients (also known as ratio ratios). OR coefficients reflected the relative effects of the independent variables in the study. Since the subjects in this paper were divided into the middle- and high-school stages, only these two learning stages were used for comparison in this study. As can be seen from Table 2, in terms of gender, girls were more likely to suffer from emotional neglect compared with boys, and girls were also more likely to develop social anxiety; in terms of only children, compared with non-only children, rural left-behind only children were more likely to engage in cyberbullying, only children were more likely to develop social anxiety, and the difference in terms of emotional neglect was not significant; in terms of learning stage, compared with high-school stage, middle-school stage students were more prone to cyberbullying.

### 3.4. Descriptive Statistical Analysis among the Main Variables

The means and standard deviations of the variables in this study and the correlation coefficients between the main variables are shown in Table 3. The results of the correlation analysis showed that the correlations between the main variables in this study all reached the level of significance (*p* < 0.01). First, adolescents’ emotional neglect was significantly and positively correlated with cyberbullying (r = 0.528, *p* < 0.01), meaning that adolescents with emotional neglect would commit cyberbullying more frequently. Second, there was a positive correlation between emotional neglect and social anxiety (r = 0.364, *p* < 0.01), and both cyberbullying and social anxiety were also significantly positively correlated (r = 0.650, *p* < 0.001), a result that paves the way for the subsequent verification of the mediating effect of social anxiety.

### 3.5. Intermediary Model Testing

**Figure 2:**
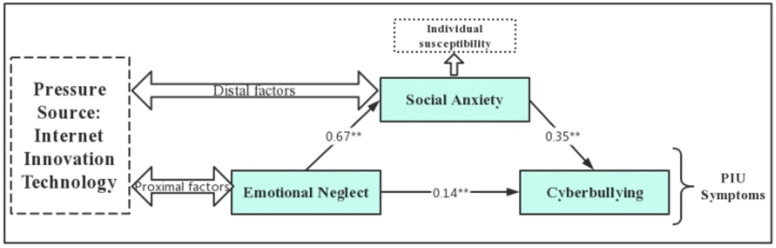
Intermediary effect diagram. Note: ** *p* < 0.01.

The data were first standardized. Amos 24.0 was used to build the mediated hypothesis model (Figure 2). The hypothesis model and its degree of fit were tested, and the analysis results are shown in Figure 2. The model fit was good: χ^2^/df = 0.871, RMSEA = 0.023, GFI = 0.987, AGFI = 0.992, NFI = 0.991, CFI = 0.994. From the model, it is clear that all direct paths reached significant levels: emotional neglect significantly and positively predicted cyberbullying (β = 0.14, *p* < 0.01); emotional neglect positively predicted social anxiety (β = 0.67, *p* < 0.01); and social anxiety positively predicted cyberbullying (β = 0.35, *p* < 0.01).

The percentile Bootstrap bias correction method was further used to reduce Type II errors [56,57]. The Bootstrap 95% confidence interval of the mediation effect was calculated by repeating the sample 5000 times. If the confidence interval did not contain 0, it indicated that the mediation effect was significant [58,59]. The analysis results are shown in Table 4. The Bootstrap 95% confidence intervals for each path did not contain 0, with 95% confidence intervals [0.618, 0.796], from which the mediation effect could be judged to hold. In this model, the total indirect effect value of social anxiety in the impact of emotional neglect on cyberbullying among rural left-behind adolescents was 0.199, where the full effect = direct effect + indirect effect = 0.756 = 0.557 + 0.199; the mediating effect share = indirect effect/total effect (%) = 0.199/0.756 = 26.32%; therefore, social anxiety was partially mediated, with a mediated share of 26.32%.

## 4. Discussion

### 4.1. The Relationship between Emotional Neglect and Cyberbullying among Rural Left-behind Adolescents

Regarding the first research question, a relationship existed between emotional neglect and cyberbullying among rural left-behind adolescents. This paper concludes that emotional neglect positively affected cyberbullying among rural left-behind adolescents. This was consistent with our expected research findings.

First, rural left-behind adolescents are minors in rural areas whose parents or guardians cannot accompany them because they are away for extended periods for work, business, farming, or other reasons, and they are forced to live alone or with their grandparents, neighbors at home, or relatives [60]. Generally, such adolescents are cared for by grandparents or other relatives, have more severe emotional neglect problems, and face multiple risks and challenges, such as psychological problems, physical health problems, and fewer educational opportunities [61]. This study focused on this particular group and examined the effects of emotional neglect on cyberbullying behavior in this group. The results of the mediating effects study concluded that the first hypothesis of this paper held, as actual and emotional neglect were positively related to cyberbullying behavior among rural left-behind youth. Second, the findings of this study are similar to those of youth bullying studies in the context of the school bullying theory because emotional neglect is often a critical factor in the long-term adverse effects of various internal and external adversities. In addition, researchers have recognized that when parents or essential family members neglect adolescents’ emotional needs, this may lead to long-term effects of negative emotions and behaviors [62,63,64]. Due to the specificity of the study population in this paper, this group of rural left-behind adolescents tended to face a more chronic lack of family presence and education. This further contributed to this group’s indulgence in the online world to gain value. Thus, we also indirectly proved that emotional neglect is a significant causal factor for cyberbullying behavior among rural left-behind adolescents.

### 4.2. The Mediating Role of Social Anxiety

Regarding the second research question, social anxiety played a role in the process of emotional neglect influencing cyberbullying among rural left-behind adolescents. The findings suggest that emotional neglect and cyberbullying are significantly related and positively predictive, while social anxiety reflects a partial mediating effect. The second hypothesis of this paper holds.

The results of this study are consistent with Adumitroaie and Hu’s findings that left-behind adolescents cannot integrate into reciprocal interpersonal relationships due to a chronic lack of opportunities and occasions; therefore, a cognitive tendency for social anxiety arises [65,66,67]. As a result, it is difficult for them to gain practice in social skills. When there is a lack of intimacy as well as emotional neglect, left-behind children cannot understand and express their emotions and feelings, leading to uneasiness and nervousness when interacting with others and social anxiety [68]. Rural left-behind adolescents are prone to loneliness and loss and lack a sense of belonging when their parents are absent for long periods. This effect can make them lack confidence in their growth and be reluctant to contact and communicate with other peers who share their hobbies, which can lead to social anxiety [69]. Some studies have pointed out that rural left-behind adolescents are more likely to develop social anxiety due to their strong Internet dependence and emotional neglect. However, the analysis in this paper also suggests that if parents or supervisors neglect the emotional needs of left-behind adolescents, leading to social anxiety, they may promote their involvement in cyberbullying. From the above research data, it can be found that the neglect of fathers’ emotional attention makes Chinese rural left-behind youth more likely to be perpetrators of cyberbullying.

### 4.3. Research Significance

This study contributes significant research value by taking Chinese rural left-behind adolescents as the research object. Cyberbullying is a globally shared social problem of adolescents, and the factors of its formation are complex, both as a projection of complex social issues at the school level and coinciding with the particular transition period of adolescents. Focusing on this problem among left-behind adolescents not only enables us to understand better the ontological causes of school bullying in this specific group but also helps us recognize and understand the causes of the problem in other adolescent groups. The inner experiences and feelings unique to adolescence are essential factors in the gradual increase of autonomy and endogenous development of adolescence. Their varying degrees of absence and deviations in access constitute the leading cause of cyberbullying among left-behind adolescents. Therefore, positive parenting and appropriate emotional guidance in schools, as well as improving the social skills of rural adolescent groups, will reduce adolescent aggression and decrease the likelihood of peer interaction and bullying problems [70,71].

Theoretically, this study answers how emotional neglect can produce cyberbullying by increasing individuals’ social anxiety, starting with cyberbullying behavior among rural left-behind adolescents. This study further expands the study of the mechanism of the relationship between emotional neglect and cyberbullying among rural left-behind adolescents. At the practical level, this study verified the effect of emotional neglect on cyberbullying behavior. Emotional neglect can influence the cyberbullying behavior of rural left-behind adolescents, which is a problem that needs to be noticed. To reduce this cyberbullying, comprehensive measures are needed, including paying attention to the mental health needs of left-behind adolescents, promoting parent–child relationships, and fostering good behavioral habits among adolescents. Supervisors and parents are also reminded that they should pay attention to the emotional needs of adolescents and actively communicate with them, thus reducing their risk of engaging in cyberbullying. The mediating role of social anxiety also suggests that classroom teachers, counselors, or mental health educators should guide rural left-behind adolescents to develop positive interpersonal concepts, improve interpersonal skills, avoid the phenomenon of social anxiety, replace online dependence with realistic interpersonal interactions, and use the Internet rationally to reduce the impact of negative psychological experiences on individuals.

## 5. Limitations

The present study still has shortcomings. First, this study’s focus on rural left-behind youth in cyberbullying was limited to individuals with social anxiety. However, the reality of cyberbullying situations is full of participants with different personalities, ranging from emotionally neglected rural left-behind adolescents with social anxiety to emotionally neglected rural left-behind adolescents without social anxiety, as well as rural left-behind adolescents who are not emotionally neglected. Therefore, these comparative studies are also a solid addition to this study in the future. Second, limited by the sensitivity and virtual nature of cyberbullying behavior, this study could not construct an actual cyberbullying situation but only explored the effects of emotional neglect and social anxiety on rural left-behind adolescents’ cyberbullying utilizing questionnaire collection and empirical modeling. Therefore, future research needs to add in-depth interviews and experimental studies of bullying behavior. Finally, this study used a self-assessment scale with self-reporting by the subjects. Even though confidentiality and authenticity were repeatedly emphasized during the testing process, the subjects’ responses to the questionnaire may have still been influenced by social expectations, which may have impacted the results. Future research could add comparative studies of cyberbullying in male versus female subjects and multivariate validation using the remaining scales as proof.

## 6. Conclusions

These findings add to the growing evidence of the importance of emotional neglect on rural adolescents’ cyberbullying behaviors and the mediating mechanisms of social anxiety. At the same time, it is essential to address the particular group of rural left-behind adolescents and how to effectively mitigate their problems of developing social anxiety and reducing cyberbullying behaviors. To sum up, to intervene in Chinese rural left-behind youth’s cyberbullying behaviors, we can start by alleviating emotional neglect and reducing social anxiety. Whether at the individual, family, or social levels, rural left-behind youth should be given more attention and love to expand healthy interpersonal interactions and reduce social anxiety while strengthening education on their online morality and establishing a correct view of online use. Therefore, families, communities, and schools should work together to improve the self-protection and coping abilities of rural left-behind youth by strengthening family education, social education, and Internet safety education to better integrate into society and reduce the occurrence of negative feedback behaviors. At the same time, all parties should also strengthen their attention and support to rural left-behind youth, pay attention to their physical and mental health issues, and let them feel the care and responsibility of society.

## Figures and Tables

**Table 1:** Variable Definition and Description.

Variable Name	Variable Definition	Mean	Std. Error
Cyberbullying	Average scores for items in the cyberbullying	2.975	1.185
Emotional neglect	Average scores for items in the emotional neglect	2.907	1.306
Social Anxiety	Average scores for items in the social anxiety	3.178	1.249
Gender	Male = 1, Female = 0	0.76	0.57
Whether the child is an only child	Only Child = 1, Non-only Child = 0	0.58	0.43
Study stage	Middle school = 1, High school = 0	0.64	0.17

**Table 2:** Regression analysis of relevant variables (n = 1429).

	Variables	OR	CI (95%)
Cyberbullying	Gender (with a male as reference)	1.135	1.101–1.792
	Whether the child is an only child (with the non–only child as a reference)	1.071 **	1.013–1.235
	Stage of study (with high school level as a reference)	2.351 **	2.011–2.791
Emotional neglect	Gender (with a male as reference)	0.918 *	0.718–1.212
	Whether the child is an only child (with the non–only child as a reference)	1.014	1.003–1.356
	Stage of study (with high school level as a reference)	0.789	0.711–1.235
Social Anxiety	Gender (with a male as reference)	1.012 ***	1.003–1.421
	Whether the child is an only child (with the non–only child as a reference)	0.991 **	0.893–1.113
	Stage of study (with high school level as a reference)	1.114	0.998–1.142

Note: *** *p* < 0.001, ** *p* < 0.01, * *p* < 0.05.

**Table 3:** Descriptive statistics and correlations (n = 1429).

	M	SD	Emotional Neglect	Cyberbullying	Social Anxiety
Emotional neglect	1.567	0.724	1		
Cyberbullying	3.145	0.821	0.528 **	1	
Social Anxiety	2.816	0.681	0.364 **	0.650 ***	1

Note: *** *p* < 0.001, ** *p* < 0.01.

**Table 4:** Direct and indirect effects.

Effect Value	Coeff	SE	T	P	LLCI	ULCI
Direct effect	0.557	0.0156	5.1789	<0.01	0.0711	0.1219
	Effect	BootSE	BootLLCI	BootULCI		
Indirect effects	0.199	0.0201	0.618	0.796		

## Data Availability

The raw data supporting the conclusions of this article will be made available by the authors without undue reservation.
